# Correspondence

**Published:** 1884-05

**Authors:** D. Genese


### CORRESPONDENCE.

TO THE EDITOR OF THE AMERICAN JOURNAL OF DENTAL SCIENCE.

*Dear Sir:*—In an examination made yesterday in company with Dr. Robinson of the dental institution at the Patent Office in Washington. We found one patented by Mr. Wyatt of Norwalk, Connecticut, for "relining rubber plates," dated February 19th, 1884.

May I ask through the dental journals on what ground Mr. Wyatt claims the invention, as the process of lining and adding to rubber, by means of dovetails and undercuts has been explained in all good works on Mechanical Dentistry for the past twenty years, and has been worked by myself and others in thousands of cases.

Any invention for the benefit of the profession deserves good support from us all, but patenting any process, under a false declaration, what it is new, simply because a patent has not been applied for before and where the supposed inventor has only taken out papers for notoriety amongst patients should be vigorously put down.

By Mr. Wyatt's claims we should all be prevented repairing rubber plates, or adding new teeth to old ones, or dovetailing a rubber base to hold a celluloid mounting. Did it stand good? But fortunately for the profession I am in possession of work made many years ago which fully illustrates his claim and therefore the profession *is at liberty* to reline, repair, or add to rubber dental plates without paying Mr. Wyatt a royalty.

D. GENESE.

